# Stabilizing ER Ca^2+^ Channel Function as an Early Preventative Strategy for Alzheimer’s Disease

**DOI:** 10.1371/journal.pone.0052056

**Published:** 2012-12-21

**Authors:** Shreaya Chakroborty, Clark Briggs, Megan B. Miller, Ivan Goussakov, Corinne Schneider, Joyce Kim, Jaime Wicks, Jill C. Richardson, Vincent Conklin, Benjamin G. Cameransi, Grace E. Stutzmann

**Affiliations:** 1 Department of Neuroscience, Rosalind Franklin University/The Chicago Medical School, North Chicago, Illinois, United States of America; 2 Department of Neuroscience, University of Connecticut, Farmington, Connecticut, United States of America; 3 Section of Neurology, The University of Chicago, Chicago, Illinois, United States of America; 4 Research & Development China, United Kingdom Group, GlaxoSmithKline, Stevenage, United Kingdom; 5 Lyotropic Therapeutics, Ashland, Virginia, United States of America; Federal University of Rio de Janeiro, Brazil

## Abstract

Alzheimer’s disease (AD) is a devastating neurodegenerative condition with no known cure. While current therapies target late-stage amyloid formation and cholinergic tone, to date, these strategies have proven ineffective at preventing disease progression. The reasons for this may be varied, and could reflect late intervention, or, that earlier pathogenic mechanisms have been overlooked and permitted to accelerate the disease process. One such example would include synaptic pathology, the disease component strongly associated with cognitive impairment. Dysregulated Ca^2+^ homeostasis may be one of the critical factors driving synaptic dysfunction. One of the earliest pathophysiological indicators in mutant presenilin (PS) AD mice is increased intracellular Ca^2+^ signaling, predominantly through the ER-localized inositol triphosphate (IP_3_) and ryanodine receptors (RyR). In particular, the RyR-mediated Ca^2+^ upregulation within synaptic compartments is associated with altered synaptic homeostasis and network depression at early (presymptomatic) AD stages. Here, we offer an alternative approach to AD therapeutics by stabilizing early pathogenic mechanisms associated with synaptic abnormalities. We targeted the RyR as a means to prevent disease progression, and sub-chronically treated AD mouse models (4-weeks) with a novel formulation of the RyR inhibitor, dantrolene. Using 2-photon Ca^2+^ imaging and patch clamp recordings, we demonstrate that dantrolene treatment fully normalizes ER Ca^2+^ signaling within somatic and dendritic compartments in early and later-stage AD mice in hippocampal slices. Additionally, the elevated RyR2 levels in AD mice are restored to control levels with dantrolene treatment, as are synaptic transmission and synaptic plasticity. Aβ deposition within the cortex and hippocampus is also reduced in dantrolene-treated AD mice. In this study, we highlight the pivotal role of Ca^2+^ aberrations in AD, and propose a novel strategy to preserve synaptic function, and thereby cognitive function, in early AD patients.

## Introduction

Existing therapies for Alzheimer’s disease (AD) target late-stage symptoms and largely delay cognitive loss rather than prevent disease progression. Recent clinical trials based on the amyloid hypothesis [1] have unfortunately failed to provide therapeutic opportunities [2]–[5]. As Aβ levels are poorly correlated with cognitive performance in AD and mild cognitive impairment (MCI) patients, alternative strategies need further exploration [6]–[8]. For example, investigating means to normalize early pathogenic cascades and preserve synaptic function may be more fruitful, as it is the loss of synaptic integrity that correlates best with, and may be a causative agent of, cognitive decline in AD [9], [10].

One candidate pathway which directly impinges on core AD features, including synaptic dysfunction, is ER Ca^2+^ signaling. In AD mouse models, increased ER Ca^2+^ release occurs prior to histopathology and memory loss [11], [12] and may serve as a critical early facilitator of AD-linked cellular pathology. Similarly, presymptomatic 3xTg-AD and TASTPM mice demonstrate exaggerated RyR-evoked Ca^2+^ release in dendrites and spines associated with altered synaptic plasticity homeostasis and network depression [13]–[16], and early increases in IP_3_R-mediated Ca^2+^ release have been found to upregulate pCREB and CAMKIV which are recruited during the induction and expression of synaptic plasticity [17]–[19]. Notably, the elevated RyR2 expression, cognitive decline, and synaptic loss observed in patients with MCI, are mirrored by increased RyR2 expression and Ca^2+^ release in presymptomatic AD mice [12], [13], [20], [21]. Increased RyR3 expression is also coincident with Aβ deposition in later stages of pathology in AD mice, possibly as a neuroprotective response [22].

Therefore, if aberrant RyR-evoked Ca^2+^ signaling is an early and integral feature of familial and sporadic AD, normalizing Ca^2+^ should be an effective strategy to halt disease progression. To test this hypothesis, we examined whether treating AD mice with the RyR-inhibitor dantrolene could stabilize Ca^2+^ signaling and synaptic transmission in AD mice while having minimal impact on these functions in normal non-transgenic mice. Previous studies demonstrated that acute dantrolene, applied in vitro, reversed aberrant ER Ca^2+^ signaling in cortical neurons from AD mice [12], [23]. More recently, long-term chronic dantrolene treatment was found to reduce cognitive deficits in aged AD mice (>12 months) and reduce Aβ levels in aged AD mouse brains and cultured neurons [24], [25]. These and related studies lend increasing support for targeting calcium signaling pathways as an effective therapeutic strategy for AD. However, here, we are proposing that stabilizing Ca^2+^ signaling earlier in the disease process has widespread beneficial effects on several Ca^2+^-dependent neuronal events that are centrally linked to the features of AD. Specifically, we show that sub-chronic dantrolene treatment (4 weeks) normalizes many of the core pathogenic features observed in presymptomatic 3xTg-AD and adult TASTPM mouse models, including dysregulated ER Ca^2+^ signaling, Aβ deposition, increased RyR2 expression levels, and synaptic transmission and plasticity abnormalities, while having little effect in NonTg controls. Based on the marked beneficial effects with relatively short treatment, we propose that the RyR is an effective target for developing disease-modifying therapeutics for AD.

## Materials and Methods

### Transgenic Mice

Two AD mouse models were used in this study: 2–3 month old 3xTg-AD (APP_SWE_, Tau_P301L_, and PS1_M146V_KI) mice described in [11]; and 5–6 month old TASTPM double-transgenic expressing PS1_M146V_ and APP_SWE_ obtained from GlaxoSmithKline R&D [26]. All transgenes are under the Thy1 promoter, with the exception of the PS1 mutation in the 3xTg-AD model, which is a knock-in and under the control of the endogenous murine PS promoter. Age-matched control non-transgenic (NonTg) mice were of the same background strain as the AD mice (J29/C57bl/6 and C57bl/6 respectively). Both male and female mice were used in this study. For clarity of presentation, we will refer to the 3xTg-AD and TASTPM mice when discussed together as AD-Tg, with the control mice referred to as NonTg. Both male and female mice were used in this study. Animals were cared for and used in accordance with protocols approved by the Rosalind Franklin University Institutional Animal Care and Use Committee and in accordance with the GSK Policy on the Care, Welfare and Treatment of Laboratory Animals.

### Dantrolene Treatment and Dose Schedule

A nanocrystal formulation of dantrolene (Lyotropic Therapeutics, Inc., [27]) was administered intraperitoneally (IP, 10 mg/kg in sterile water) to AD-Tg and NonTg mice at two time points: (1) daily injections for 4 weeks starting at 2 months of age for the 3xTg-AD mice (corresponding to plaque-free stage); and (2) daily injections for 4 weeks starting at 5 months for the TASTPM (coinciding with moderate plaque formation and onset of cognitive deficits; [26], [28]. Control mice were administered 0.9% saline daily.

### Hippocampal Slice Preparation

In brief, mice were deeply anesthetized with halothane and rapidly decapitated. The brains were extracted rapidly and 300 or 400 µm-thick transverse hippocampal slices were cut (for either patch clamp or field potential recording, respectively) with a vibrating microtome (Campden Instruments) into ice-cold oxygenated artificial cerebrospinal fluid (aCSF) with the following composition (in mM): 125 NaCl, 2.5 KCl, 1.25 KH_2_PO_4_, 1.2 MgSO_4_, 2 CaCl_2_, 10 Dextrose and 25 NaHCO_3_ [13]. Intact slices were placed in a holding chamber containing aCSF at room temperature (27°C) and oxygenated with 95% O_2_–5% CO_2_.

### Extracellular Field Potential Recordings and Drug Treatment Protocols

To record extracellular field potentials, slices were transferred to an interface chamber (Harvard Apparatus) and perfused with oxygenated aCSF (1.5 ml/min) at room temperature (27°C) and covered with a continuous flow of humidified gas (95% O_2_–5% CO_2_). Data were acquired at 10 kHz using pClamp 9.2 software with an AxoClamp 2B amplifier and a Digidata 1322A board for digitization (Molecular Devices). Field EPSPs (fEPSPs) were recorded in the stratum radiatum of the CA1 subfield of the hippocampus using recording microelectrodes (2–6 MΩ) filled with aCSF. Microelectrodes were pulled from glass capillaries (Harvard Apparatus) on a P-2000 pipette puller (Sutter Instruments). Synaptic responses were evoked by stimulation of the Schaffer collateral/commissural pathway with a bipolar stimulating electrode. Baseline fEPSPs were evoked at 30% of the maximum fEPSP at 0.05 Hz for 20 minutes before the induction of LTP. LTP was induced using high frequency stimulation (2 trains at 100 Hz for one second separated by a 10 s interval) at the baseline stimulus intensity. fEPSPs were then recorded for 1 hour at 0.05 Hz after the induction of LTP. For experiments with dantrolene (10 µM), a 20 minute baseline at 0.05 Hz was recorded in aCSF, after which dantrolene was perfused continuously over the slice. The same stimulus intensity was used to record a 20 minute baseline in dantrolene, after which LTP was induced, followed by 1 hour recording in the continued presence of dantrolene. Input/Output (I/O) curves were generated using stimulus intensities from 0 to 225 µA in increments of 25 µA. Paired pulse facilitation (PPF) was assessed using an interstimulus interval of 50 ms. Ten successive paired responses were recorded at 0.05 Hz. Electrophysiological recordings were measured offline using pClamp 9.2 software and analyzed using Origin Pro8 and Microsoft Excel software. For LTP, fEPSP slopes were expressed as a percentage of the average slope from the 20 minute baseline recordings. For PPF, fEPSP amplitudes were expressed as a ratio of the second response over the first. For I/O curves, the first 5 ms of the fEPSP slope was measured. Data are expressed as mean±SEM and assessed for significance using Student’s two-tailed *t* test or one-way or two-way ANOVA with Scheffe *post hoc* analysis, where *n* denotes the number of slices examined in extracellular field experiments.

### Patch-clamp Electrophysiology

Hippocampal brain slices (300 µm) were superfused at 2 ml/min with aCSF equilibrated with 95% O_2_/5% CO_2_ at room temperature. Patch pipettes (5 MΩ) were filled with intracellular solution containing the following substances (in mM): 135 K-gluconate, 2.0 MgCl_2_, 4.0 Na_2_ATP, 0.4 NaGTP and 10 Na-phosphocreatine, 10 HEPES (pH adjusted to 7.3 with KOH, and 50 µM fura-2 (Invitrogen). Hippocampal CA1 pyramidal neurons were identified visually via infrared differential interference contrast optics, and electrophysiologically by their passive membrane properties and spike frequency accommodation. Membrane potentials were obtained in current-clamp mode acquired at 10 kHz with a Digidata 1322 A-D converter and MultiClamp 700B amplifier, and were recorded and analyzed using pClamp 10.2 (Molecular Devices). Series resistance was monitored throughout the experiment and drifts beyond 10 MΩ were discarded.

### Ca^2+^ Imaging

Ca^2+^ imaging within individual neurons was performed in brain slice preparations using a custom-made video-rate multiphoton-imaging system based on an upright Olympus BX51 microscope frame [29]. Individual neurons were filled with the Ca^2+^ indicator, bis-fura-2 (50 µM) via the patch pipette. Laser excitation was provided by 100 fs pulses at 780 nm (80 MHz) from a Ti:sapphire laser (Mai Tai Broadband, Spectra-Physics). The laser beam was scanned by a resonant galvanometer (General Scanning Lumonics), allowing rapid (7.9 kHz) bidirectional scanning in the x-axis, and by a conventional linear galvanometer in the y-axis, to provide a full-frame scan rate of 30 frames/s. The laser beam was focused onto the tissue through an Olympus 40x water-immersion objective (numerical aperture 0.8). Emitted fluorescence light was detected by a wide-field photomultiplier (Electron Tubes) to derive a video signal that was captured and analyzed by Video Savant 5.0 software (IO Industries). Further analysis of background-corrected images was performed using MetaMorph software. For clarity, results are expressed as inverse ratios so that increases in [Ca^2+^] correspond to increasing ratios. The % change is calculated as [(F_0_/ΔF)−1]*100 where F_0_ is the average resting fluorescence at baseline and ΔF is the decrease of fluorescence reflecting Ca^2+^ release. Differences between drug- and saline-treated groups were assessed using two-way ANOVA and Scheffe *post hoc* analysis for significance (p<0.05). For data sets measuring somatic Ca^2+^ responses, the nucleus was excluded.

### Immunohistochemistry and Aβ Deposition

Mice were transcardially perfused with ice-cold PBS (3 ml) followed by 4% paraformaldehyde (5 ml). Brains were extracted and fixed overnight in 30% sucrose-cryoprotectant solution. Coronal hippocampal sections 40 µm thick were cut on a cryostat and collected in TBS (0.1 M Tris, 0.9% saline, pH 7.4).

Thioflavin S Staining: Free-floating hippocampal sections were washed with TBS (4×3 minutes). The sections were soaked in 0.5% thioflavin S (in 50/50 ethyl alcohol/distilled water, Sigma-Aldrich) for 10 minutes. This was followed by 2×3 minute washes with 50% ethyl alcohol. Sections were washed again with TBS (2×3 minutes), mounted with minimal drying, and coverslipped with anti-fade mounting medium PVA-DABCO for microscopy.

4G8 Staining: Free-floating hippocampal sections were washed with TBS (3×10 minutes) followed by a 10 minute wash with 70% formic acid. This was followed by a 10 minute wash with TBS+ (TBS +0.1% Triton-X) followed by TBS (3×10 minutes). Tissue sections were blocked with 10% goat serum in TBS for 1–3 hours, then incubated with primary antibody (4G8, 1∶1500, Covance) diluted in TBS++ (TBS +3% goat serum +0.1% Triton-X) for 72 hours at 4°C. The sections were washed again with TBS (3×10 minutes) and incubated in secondary antibody (Alexa Fluor 488 goat anti-mouse, 1∶250, Invitrogen) diluted in TBS++ for 24 hours at 4°C. This was followed by 3×10 minute washes with TBS. Sections were mounted with minimal drying and coverslipped with anti-fade mounting medium PVA-DABCO for microscopy.

Confocal images of immunolabeled tissue were obtained using 4X and 10X objective lenses on the Olympus Fluoview confocal microscope. Density of amyloid plaques was quantified by averaging the percent area staining positive (thresholded above background staining, as determined by software parameters and experimenter confirmation) within the hippocampus and cortex from 3–5 sections from each experimental animal using MetaMorph Software (Molecular Devices). There were no significant differences in the intensity of background threshold values across animal strains or treatment conditions (p>0.05). The experimenter was blind to animal strain and treatment condition. Density analysis did not distinguish between the number of plaques or the size of individual plaques.

### Body Weights and Heart Weights

Mice were perfused with paraformaldehyde as above. The heart was extracted and any fluid remaining within the organ was removed. The heart was weighed and the ratio of body: heart weights was calculated (Table 1). Heart and body weights were not significantly different between saline or dantrolene-treated NonTg and 3xTg-AD mice (one-way ANOVA, body weights: p>0.05; heart weights: p>0.05; n = 4 for all groups). Body weights were obtained for the TASTPM mice, and were also not different between treatment groups, or from the NonTg and 3xTg-AD groups (p>0.05).

**Table 1:** Ratio of heart and body weights from control and dantrolene-treated mice.

Group	Body Weight (g)	Heart Weight (g)	HW:BW
**NonTg Saline (4)**	19.3±1.9	0.084±0.006	4.4×10^−3^
**NonTg Dantrolene (4)**	20.5±2.9	0.091±0.015	4.4×10^−3^
**3xTg-AD Saline (4)**	22.5±2.3	0.091±0.006	4.05×10^−3^
**3xTg-AD Dantrolene (4)**	22.8±2.0	0.096±0.009	4.25×10^−3^

Table summarizes the effects of sub-chronic dantrolene treatment on heart and body weights of NonTg and 3xTg-AD mice.

### RNA Extraction and Quantitative RT-PCR

Total mouse hippocampal RNA was extracted and purified from 20–40 mg samples (wet weight) dissected from AD-Tg and NonTg animals using TRI Reagent and the corresponding protocol (Invitrogen). RNA was constituted in sterile, nuclease-free water, and sample concentrations were determined spectrophotometrically at OD_260 nm_. After DNase treatment of 1 µg of total RNA (DNAfree kit; Ambion), dNTP and random primers were used for cDNA synthesis using the High Capacity Reverse Transcription kit (Applied Biosystems) and associated protocol.

1 µl of the resulting cDNA products was evaluated using real-time PCR. Target genes were amplified and evaluated using the 7500 RT PCR System and SYBR green detection (Applied Biosystems). Cycling parameters were as follows: 2 min at 50°C, 10 min at 95°C, and then 40 cycles at 95°C for 15 s, followed by 60°C for 60 s. A dissociation phase was added to the end of each cycle to determine product purity. Oligonucleotide primers were synthesized by Invitrogen for target gene amplification. Cyclophilin A was used as an endogenous control gene. Primer sequences are as follows: *RyR1*: forward (F), 5′-TCTTCCCTGCTGGAGACTGT- 3′; reverse (R), 5′-GTGGAGAAGGCACTTGAGG- 3′; *RyR2*: (F), 5′-TCAAACCACGAACACATTGAGG-3′; (R), 5′-AGGCGGTAAAACATGATGTCAG-3′; *RyR3*: (F), 5′-TGGCCATCATTCAAGGTCT-3′; (R), 5′-GTCTCCATGTCTTCCCGTA-3′; *CycloA*: (F), 5′-GGCCGATGACGAGCCC-3′; (R), 5′-TGTCTTTGGAACTTTGTCTGCAAAT-3′. Primer specificities were validated by the presence of a single amplicon for each primer set at the predicted sizes (95, 125, and 83 bp for RyR1, RyR2, and RyR3, respectively) after agarose gel electrophoresis of PCR products and ethidium bromide detection. Product purity was supported by the presence of a single peak present on dissociation/melt curves for each primer set. Each sample was evaluated in triplicate. Amplification data were analyzed using the comparative cycle threshold (ΔΔCt) method after normalization to Cyclophilin A and are represented as mean±SEM. A one-way ANOVA was used to determine statistical significance.

## Results

### Dantrolene Treatment Reverses Intracellular Ca^2+^ Signaling Dysregulation in AD Mice

One of the earliest detectable changes in neuronal signaling in the 3xTg-AD and TASTPM mice is a marked increase in ER Ca^2+^ release, which has been associated with AD-linked pathology ranging from synaptic impairment to increased amyloid deposition [13]–[16], [30]. The IP_3_R-mediated Ca^2+^ responses contribute to this phenomenon within the soma, while the RyR is a significant contributor of aberrant Ca^2+^ release within the dendritic and synaptic compartments in addition to the soma [17], [18], [31]. Here we measured evoked Ca^2+^ -responses in CA1 pyramidal neurons from both the soma (nucleus excluded) and dendrites via activation of voltage-gated Ca^2+^ channel (VGCC) during a spike train and intracellular release elicited by application of the RyR agonist caffeine (20 mM for 1 min). Passive membrane properties, including resting membrane potential (Vm) and input resistance (Rin), for the cells used in these studies are shown in Table 2, with no significant differences between dantrolene versus saline treatment groups nor between AD-Tg and NonTg mice.

**Table 2:** Electrophysiological properties of hippocampal pyramidal neurons from control and dantrolene-treated mice.

Group	Vm (mV)	Rin (MΩ)
**NonTg Saline (10)**	−72±0.1	154±11
**NonTg Dantrolene (10)**	−71±0.3	156±14
**TASTPM Saline (6)**	−71±0.5	162±10
**TASTPM Dantrolene (6)**	−69±0.6	161±15
**3xTg-AD Saline (5)**	−70±0.4	157±12
**3xTg-AD Dantrolene (14)**	−71±0.5	156±10

Table summarizes the effects of chronic dantrolene treatment on resting membrane potential (Vm) and input resistance (Rin) of hippocampal pyramidal neurons from NonTg and AD-Tg mice.

**Figure 1:**
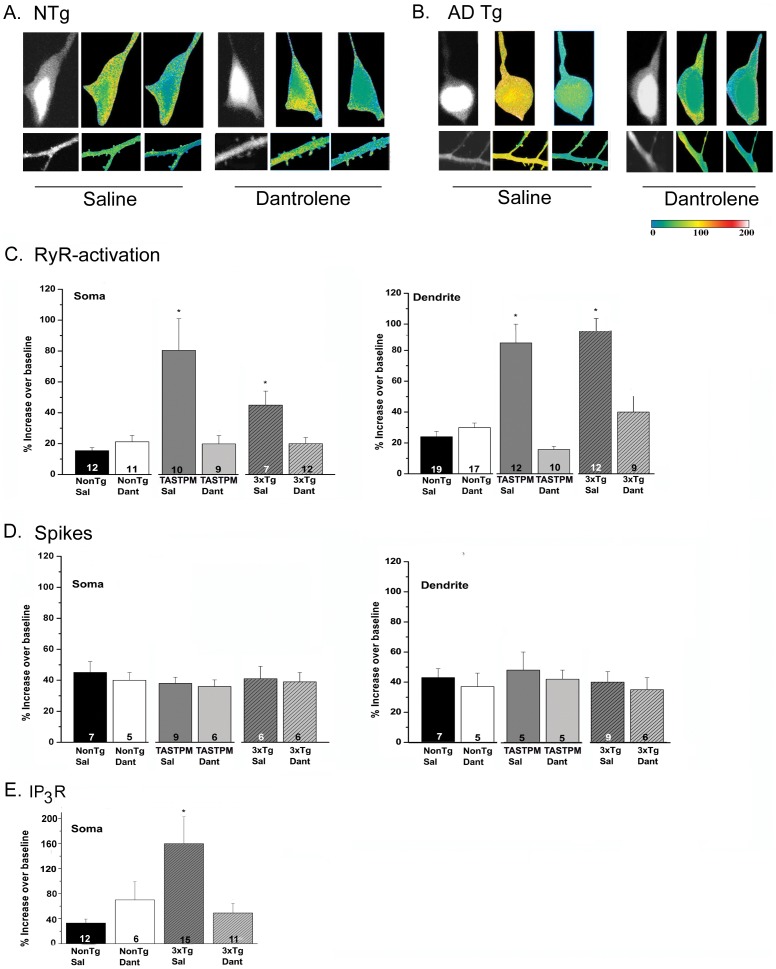
Sub-chronic dantrolene treatment normalizes aberrant ER Ca^2+^ signaling in AD-Tg neurons. 2-photon Ca^2+^ images of representative CA1 pyramidal neurons showing that sub-chronic dantrolene treatment in AD-Tg neurons returns the RyR-evoked Ca^2+^ signals back to NonTg control levels. Ca^2+^ signals were evoked by caffeine, a RyR agonist. *(* ***A, B*** *)* Dantrolene treatment reduces intracellular Ca^2+^ release evoked through RyR stimulation in pyramidal neuron soma and dendrite. Representative fura-2 images of hippocampal CA1 pyramidal neuron somata (top) and dendrites (bottom) are shown under resting (baseline), peak response to caffeine (20 mM applied for 1 min), and recovery (wash) conditions for slices from *(* ***A*** *)* saline-treated (left) and dantrolene-treated (right) NonTg and *(* ***B*** *)* saline-treated (left) and dantrolene-treated (right) 3xTg-AD mice. Color code bar on bottom right corresponds to soma and dendritic images. *(* ***C*** *)* Bar graphs comparing averaged maximal Ca^2+^ changes in somata (left) and dendrites and dendritic spines (right) between NonTg and AD-Tg pyramidal neurons. Averaged data show that saline-treated AD-Tg neurons have significantly larger ER Ca^2+^ transients compared with NonTg groups (both saline and dantrolene treated) and their respective dantrolene-treated groups (* = p<0.05), while dantrolene-treated AD-Tg neurons are not statistically different from NonTg controls (p>0.05). Dantrolene had no significant effect on the Ca^2+^ response in NonTg neurons. *(* ***D*** *)* Ca^2+^ influx elicited by spike trains was similar in AD-Tg and NonTg mice and was not affected by dantrolene treatment. *(* ***E*** *)* In dantrolene-treated 3xTg-AD mice, somatic IP_3_-evoked Ca^2+^ responses are normalized to within NonTg levels, and are significantly reduced compared to the 3xTg-AD saline-treated animals. Values are shown as mean ± SEM with sample number indicated within each bar.

In this study and in others, RyR- Ca^2+^ responses evoked by caffeine, but not by spike trains, were elevated in 3xTg-AD and TASTPM mice compared to control mice [13]–[15]. Here, we demonstrate that sub-chronic dantrolene treatment normalized the ER Ca^2+^ response in hippocampal pyramidal neurons in the AD-Tg mice such that Ca^2+^ signaling was returned to levels observed in both saline- and dantrolene-treated NonTg mice (p>0.05; Figure 1). In contrast, the Ca^2+^ response to VGCC activation was not elevated in the AD-Tg mice, and neither AD-Tg nor NonTg VGCC Ca^2+^ responses were affected by dantrolene treatment. Our earlier research demonstrates positive feed-forward interactions between the IP_3_R and RyR in 3xTg-AD mice [12], such that Ca^2+^ released from the IP_3_R receptor was sufficient to trigger a CICR response through the RyR**.** In this study as well, IP_3_R-mediated Ca^2+^ release via photolysis of caged IP_3_ is enhanced in 3xTg-AD mice and was restored to NonTg levels with sub-chronic dantrolene treatment (Figure 1, p<0.05). By stabilizing RyR function and expression, sub-chronic dantrolene treatment is likely suppressing this aberrant CICR effect initiated through IP_3_R-mediated Ca^2+^ release.

Dendritic Ca^2+^ responses in stratum radiatum were also measured in saline- and dantrolene-treated NonTg and AD-Tg mice. As shown in Figure 1, in hippocampal CA1 pyramidal neuron dendrites, the exaggerated Ca^2+^ response to caffeine in 3xTg-AD mice was reduced by dantrolene treatment, normalizing the Ca^2+^ response to that seen in NonTg mice. At the same time, dantrolene treatment had no significant effect in the NonTg controls. Dendritic action potential-evoked Ca^2+^ responses in AD-Tg mice were not different from NonTg, and were not affected by dantrolene treatment. The number of neurons measured within each group is presented within the bar graph.

### RyR2 Levels in AD Mice are Restored to NonTg Control Levels with Dantrolene Treatment

**Figure 2:**
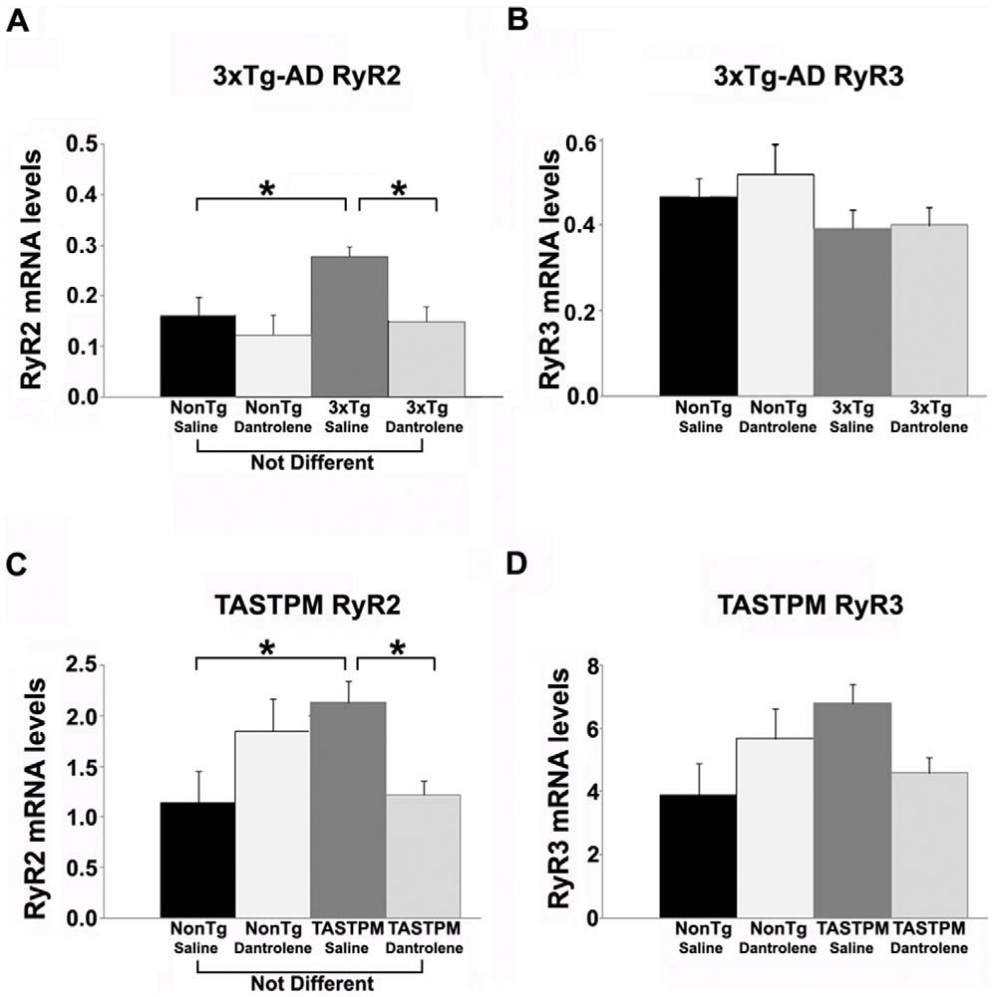
Sub-chronic dantrolene treatment normalizes expression levels of RyR2 in AD-Tg mice. Bar graphs show relative mRNA expression levels of the RyR2 *(* ***A, C*** *)* and RyR3 *(* ***B, D*** *)* isoforms from the hippocampus of NonTg and AD-Tg mice treated with 0.9% saline or 10 mg/kg dantrolene (i.p.) for 4 weeks. mRNA levels are relative to control cyclophilin A levels. Sub-chronic dantrolene treatment normalized RyR2 expression in both AD-Tg mouse strains relative to the NonTg saline- and dantrolene treated mice, and significantly reduced RyR2 levels relative to their own saline-treated AD strain. * = significantly different from saline-treated, p<0.05, n = 4–6 mice per group.

At early disease stages, the RyR2 isoform is specifically upregulated; this has been observed in human MCI patients, as well as 3xTg-AD and TASTPM mice at presymptomatic stages [13], [15], [32]. It is possible that increased RyR2 expression may contribute to the enhanced Ca^2+^ responses in AD-Tg mice and human patients. Therefore, in this study, we explored whether sub-chronic dantrolene treatment would affect RyR isoform expression in AD-Tg and NonTg mice. We found that both 3xTg-AD and TASTPM mice treated 4 weeks with dantrolene had RyR2 mRNA levels that were no different from saline-treated NonTg mice, and were significantly lower than the saline-treated AD-Tg mice of their respective strain (F _(3,22)_ = 10.6; p<0.05: F_(3,18)_ = 4.15; p<0.05 for 3xTg-AD and TASTPM respectively). Thus, dantrolene treatment restored normal levels of RyR2 expression in the AD-Tg mice (Figures 2A and 2C). RyR3 mRNA expression was not affected in 3xTg-AD mice relative to NonTg controls, and sub-chronic dantrolene treatment did not alter this (Figure 2B, p>0.05). In TASTPM mice, a similar pattern was observed, but there was a detectable trend towards increased RyR3 mRNA expression in the saline-treated TASTPM mice (Figure 2D, p = 0.07) that was reduced with dantrolene treatment. Increased RyR3 levels have been reported to occur coincident with amyloid deposition in AD mouse models, and our results are consistent with this and the presence of significant amyloid deposits in TASTPM but not 3xTg-AD mice at 6 months of age [22]. RyR1, although present in the brain at relatively low levels, does not appear to have altered protein or mRNA levels in the AD models studies, so we therefore did not analyze this isoform in this study (13).

### Dantrolene Treatment Restores Synaptic Transmission and Plasticity Homeostasis in 3xTg-AD Mice

Our previous studies in pre-symptomatic 3xTg-AD mice demonstrated disruptions in Ca^2+^-regulated synaptic transmission and plasticity mechanisms, where the RyRs are a dominant and aberrant modulator of basal synaptic transmission and presynaptic plasticity, and acute RyR inhibition *in vitro* results in a shift towards synaptic depression [13], [16]. Therefore, in the present experiments, we explore whether sub-chronic dantrolene treatment could reverse these aberrations in synaptic physiology, displaying data primarily from the 3xTg-AD mouse as this model has been characterized extensively in synaptic transmission and plasticity experiments. However, the TASTPM mice exhibit nearly identical patterns of ‘below the radar’ deficits in synaptic transmission (data not shown).

**Figure 3:**
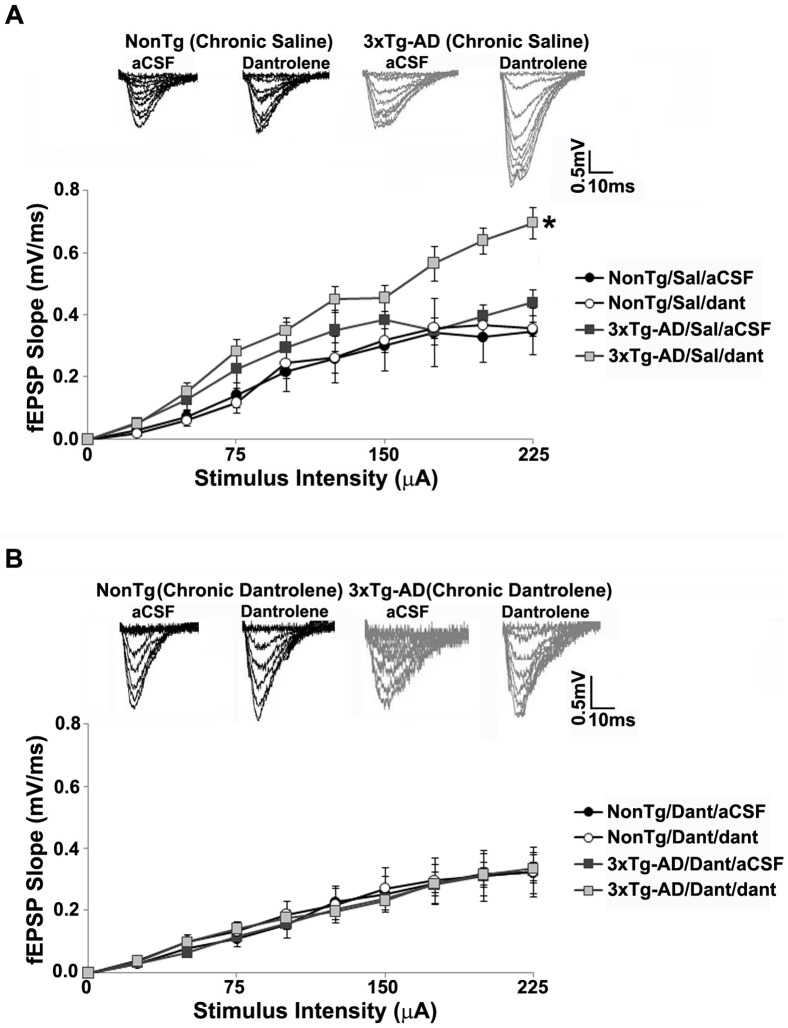
Effect of sub-chronic dantrolene treatment on synaptic strength in 3xTg-AD mice. *(* ***A–B*** *)* I/O function shows changes in fEPSP slope with increasing stimulus intensity (0–225 µA) from: *(* ***A*** *)* Saline-treated NonTg (n = 5) and 3xTg-AD (n = 5) mice with and without bath application of 10 µM dantrolene; *(* ***B*** *)* Sub-chronic dantrolene-treated NonTg (n = 10) and 3xTg-AD (n = 6) mice with and without bath application of 10 µM dantrolene; Insets *(* ***A–B*** *)* show representative fEPSP traces from NonTg and 3xTg-AD mice for each condition. * = significantly different after bath application of 10 µM dantrolene, p<0.05, n denotes number of slices.

**Figure 4:**
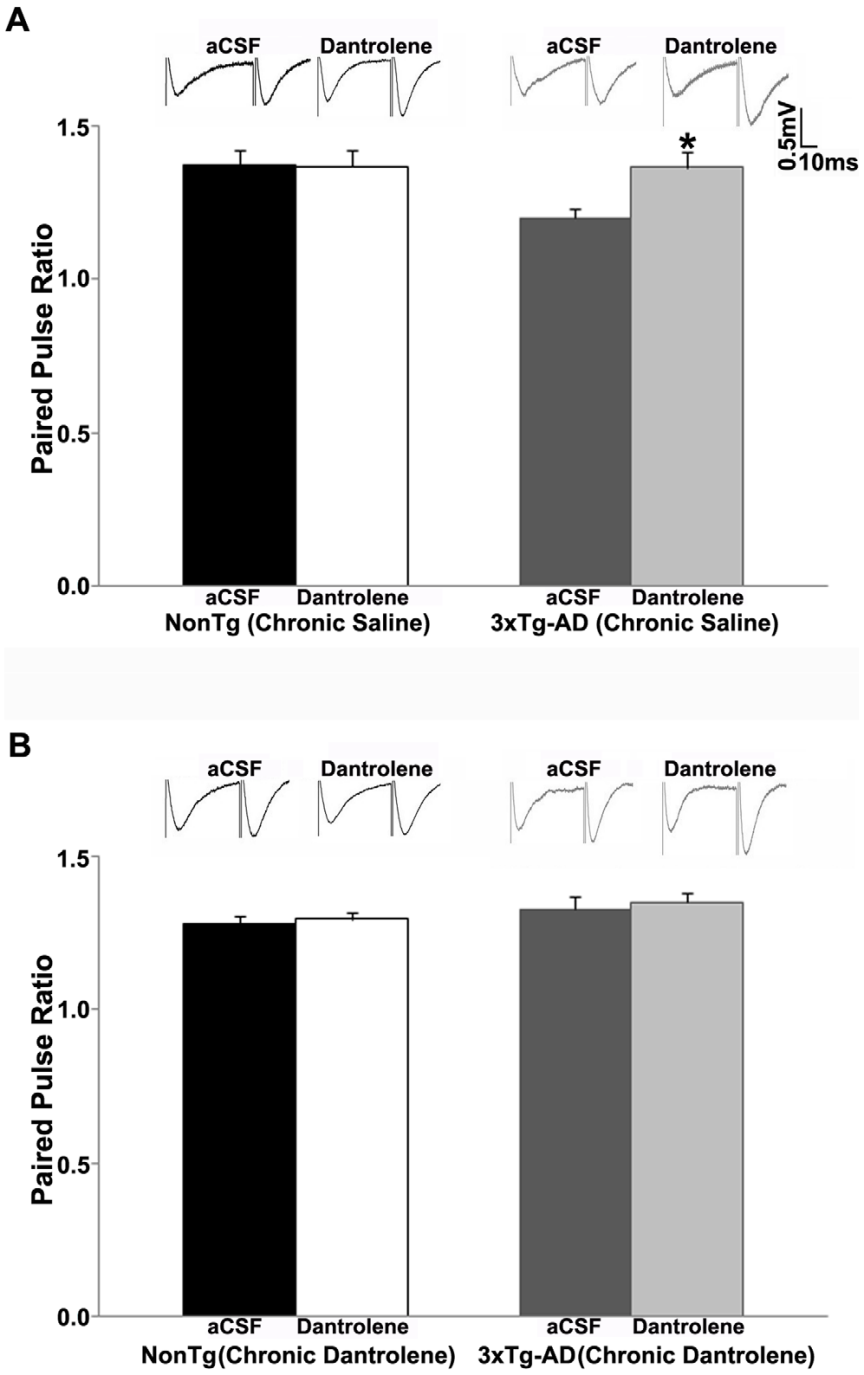
Sub-chronic dantrolene treatment normalizes PPF in 3xTg-AD mice. PPF was measured at an interstimulus interval of 50 ms. *(* ***A–B*** *)* Bar graphs show paired pulse ratio from: *(* ***A*** *)* Saline-treated NonTg (n = 5) and 3xTg-AD (n = 8) mice with bath application of 10 µM dantrolene; *(* ***B*** *)* Sub-chronic dantrolene-treated NonTg (n = 12) and 3xTg-AD (n = 9) mice with bath application of 10 µM dantrolene; Insets *(* ***A–B*** *)* show representative fEPSP traces from NonTg and 3xTg-AD mice for each condition. * = significantly different after 10 µM dantrolene bath application, p<0.05, n denotes number of slices.

We measured basal synaptic transmission and synaptic strength using Input/Output (I/O) curves. As with our previous observations, acute treatment with dantrolene (10 µM) *in vitro* had no significant effects in NonTg mice. Synaptic strength was not altered by bath application of dantrolene in saline-treated or sub-chronic dantrolene-treated NonTg mice (p>0.05, Figures 3A and 3B). As expected, bath application of dantrolene significantly increased the I/O function in saline-treated 3xTg-AD mice (t _(1, 7)_ = −5.07, p<0.05, Figure 3A). However, in the 3xTg-AD mice, sub-chronic dantrolene treatment normalized the I/O function to the NonTg character, where acute dantrolene application had little effect (p>0.05, Figure 3B). We examined the effect of sub-chronic dantrolene treatment on presynaptic plasticity by measuring paired pulse facilitation (PPF). We previously demonstrated that bath application of dantrolene increased PPF in 3xTg-AD mice, with little effect in NonTg mice. Similarly, acute application of dantrolene had little effect on PPF in saline-treated or sub-chronic dantrolene-treated NonTg mice (p>0.05, Figures 4A and 4B). PPF was increased in saline-treated 3xTg-AD mice (t _(1, 7)_ = −2.63, p<0.05, Figure 4A) with acute RyR inhibition, while sub-chronic dantrolene treatment completely reversed the RyR-mediated increases in PPF in these mice (p>0.05, Figure 4B), normalizing this response to that of NonTg mice.

**Figure 5:**
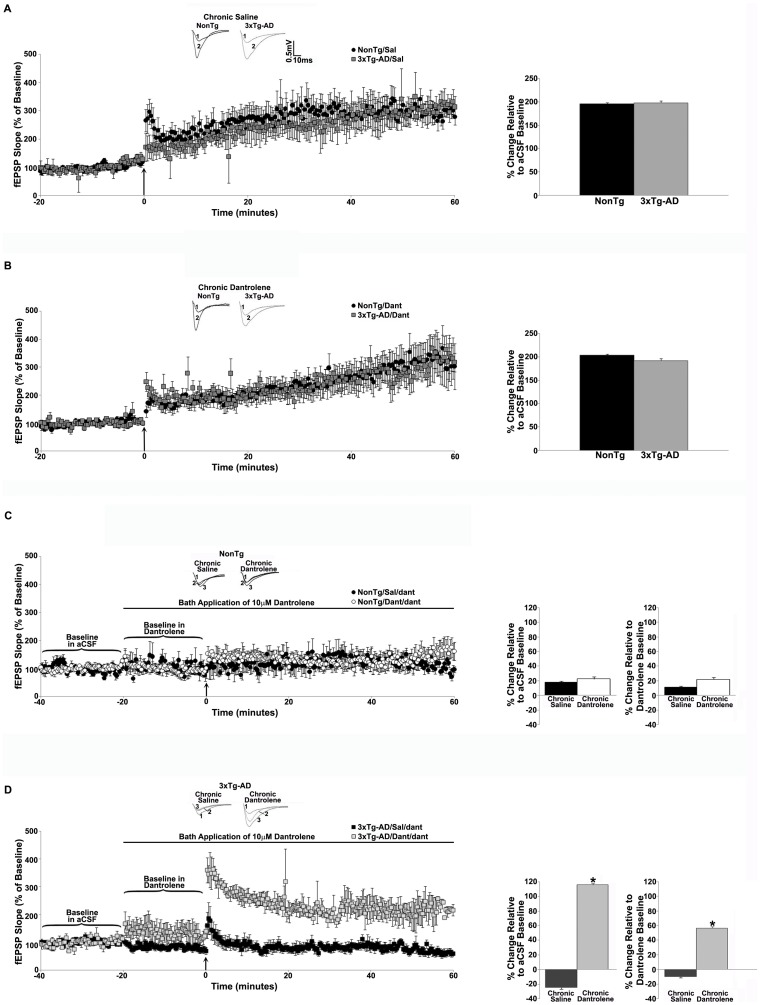
Sub-chronic dantrolene treatment rescues LTP on RyR inhibition in 3xTg-AD mice. *(* ***A–B*** *)* Left, graphs show averaged time course of LTP from NonTg and 3xTg-AD mice that were given daily injections of 0.9% saline (***A***, n = 7 for NonTg, n = 7 for 3xTg-AD) or 10 mg/kg dantrolene (***B***, n = 10 for NonTg, n = 14 for 3xTg-AD) for 4 weeks. Insets (above) show representative fEPSP traces before (1) and after (2) tetanus from NonTg and 3xTg-AD mice. Bar graphs on right show averaged % change in post-tetanus responses relative to baseline from NonTg and 3xTg-AD mice. *(* ***C–D*** *)* Left, graphs show averaged time course of LTP from NonTg mice *(* ***C*** *)* injected with saline (n = 5) or dantrolene (n = 12) with bath application of 10 µM dantrolene, and 3xTg-AD mice *(* ***D*** *)* injected with saline (n = 8) or dantrolene (n = 7) with bath application of 10 µM dantrolene. Insets (above) show representative fEPSP pre-tetanus traces before (1) and after (2) bath application of 10 µM dantrolene and post-tetanus fEPSP traces (3) with 10 µM dantrolene. Bar graphs on right show averaged % change in post-tetanus responses relative to baseline in aCSF or dantrolene from NonTg and 3xTg-AD mice. Baseline fEPSPs were recorded for 20 min at 0.05 Hz before and for 60 min at 0.05 Hz after LTP induction. The arrow indicates the time of tetanus. * = significantly different after 10 µM dantrolene bath application, p<0.05, n denotes number of slices.

Our previous studies demonstrated opposing roles of RyR-mediated Ca^2+^ stores in long-term synaptic plasticity measured in 3xTg-AD versus NonTg mice under conditions of acute RyR inhibition (13, 16). Bath application of dantrolene decreased baseline responses and shifted expression of LTP to modest LTD in 3xTg-AD mice, whereas in NonTg mice, acute dantrolene did not affect baseline responses and LTP was markedly diminished. In the present studies we were interested in the longer-term effects of RyR-stabilization when dantrolene is given sub-chronically. Under this treatment regimen, when dantrolene or saline was administered for 4 weeks, LTP was similar in the saline-treated (p>0.05, Figure 5A) and dantrolene-treated (p>0.05, Figure 5B) NonTg and 3xTg-AD mice under control aCSF conditions. We next determined whether the sub-chronic dantrolene treatment reversed the LTP disruptions in 3xTg-AD mice generated by acute inhibition of the RyR as previously described. As in the earlier studies, we compared the degree of LTP against both a pre-tetanus baseline in aCSF and pre-tetanus baseline in dantrolene. Similar to previously reported observations, acute RyR inhibition did not affect baseline responses in either saline-treated (p>0.05) or dantrolene-treated NonTg mice (p>0.05, Figure 5C). LTP was vastly reduced in both NonTg treatment groups when compared with aCSF and dantrolene baseline responses. In saline-treated 3xTg-AD mice exposed to acute dantrolene, baseline responses were reduced (16.6±1.0% below aCSF baseline, t_(1, 7)_ = 12.3, p<0.05, Figure 5D) and modest LTD expressed when compared with the control aCSF baseline (−25.1±1.6%). When measured against the acute dantrolene baseline, LTP expression was impaired, with a trend towards depression. However, in the sub-chronic dantrolene treated 3xTg-AD mice, entirely different baseline and plasticity patterns emerge. In these mice, the above-described LTP deficits were rescued, with normalized levels of LTP expression when compared with aCSF baseline (115.8±2% over baseline, Figure 5D) with maintenance of potentiation (as opposed to depression) when compared to the dantrolene baseline (56.4±2% over baseline).

### Amyloid Deposits are Reduced in Dantrolene-treated TASTPM Mice

**Figure 6:**
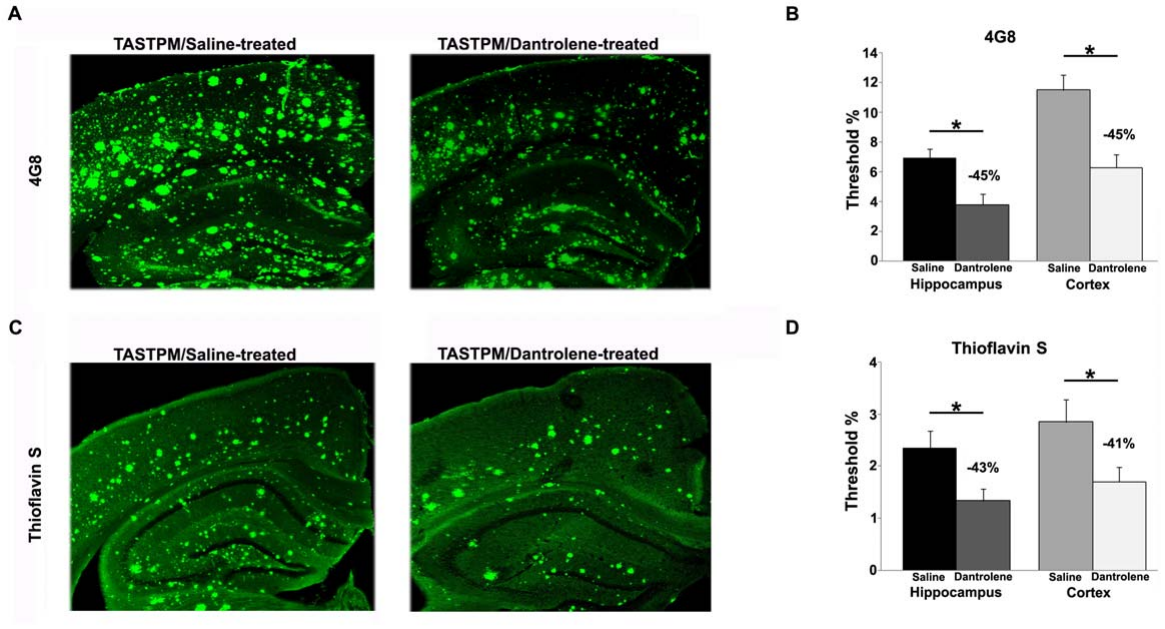
Dantrolene treatment reduces amyloid load in AD mice. *(* ***A*** *)* Representative 4G8-immunostained tissue from saline-treated (left) and dantrolene-treated (right) 6-month old TASTPM mice demonstrate an approximate 45% reduction in amyloid density with dantrolene treatment. *(* ***B*** *)* Averaged bar graphs show significant reduction in amyloid peptide staining in both the hippocampus (left) and cortex (right) in the dantrolene-treated mice compared to saline-treated mice. *(* ***C*** *)* Representative thioflavin S stained tissue from saline-treated (left) and dantrolene-treated (right) 6-month old TASTPM mice show an approximate 42% reduction in amyloid density with drug treatment. *(* ***D*** *)* Averaged bar graphs show significant reduction in the density of dense core amyloid plaques in both the hippocampus (left) and cortex (right) in the dantrolene-treated mice (white bars) compared to saline-treated mice (black bars). * = p<0.05, scale bar = 250 µm, n = 4–6 mice per group.

Circular, feed-forward interactions exist between neuronal Ca^2+^ dysregulation and amyloid deposition [30], [33]–[38], such that amyloid species can increase and destabilize Ca^2+^ signaling, while increased Ca^2+^, particularly through the ER, can facilitate amyloid aggregation. Here, we wished to test the hypothesis that RyR-mediated Ca^2+^ dysregulation can increase amyloid deposition, so therefore normalizing ER Ca^2+^ signaling can slow this cycle and result in reduced amyloid staining in hippocampal and cortical regions. We focused on TASTPM mice in these experiments as they develop amyloid aggregates and depositions in a consistent and well-documented pattern over brain regions and time [26], [28]. We measured the density of beta amyloid peptides using the 4G8 antibody which recognizes residues 18–22 of beta amyloid, and separately, we measured and compared the density of insoluble dense core amyloid plaques using thioflavin-S staining in the cortex and hippocampus of 6-month old TASTPM mice treated with dantrolene or saline. In both brain regions, we found a consistent 41–45% reduction of amyloid in the dantrolene-treated mice, for both the 4G8 and thioflavin-S stained tissue (4G8 hippocampus: t_(1, 23)_ = 3.2, p<0.05; 4G8 cortex, t_(1, 24)_ = 3.9; p<0.05; thioflavin-S hippocampus: t_(1, 33)_ = 2.5; p<0.05; thioflavin-S cortex: t_(1,22)_ = 2.3; p<0.05, Figure 6).

## Discussion

Sustained Ca^2+^ dysregulation is incorporated into many aspects of AD pathology, as both an early component contributing to synaptic pathology, and a later accelerant of amyloid and tau deposition [30], [35], [36], [39]–[41]. And recently, there has been increasing consideration given to targeting these Ca^2+^ sources as a therapeutic strategy for AD treatment. Oules et al., 2012 (25) and Peng et al., 2012 (24) demonstrate a marked improvement in cognitive function and amyloid load in AD mice with chronic systemic treatment with dantrolene, and experiments in neuronal cell cultures exposed to dantrolene suggest that the reduced amyloid is likely mediated by reductions in APP phosphorylation and β- and γ-secretase activity (25). However, using a different long-term oral dantrolene-treatment schedule (feeding twice/week, 5 mg/kg, from 2–8 months of age), Zhang et al., 2010 (2) describe an increase in amyloid load and neuronal atrophy. While the reasons for this discrepancy remain unclear, there are marked differences in the route and duration of dantrolene treatment, as well as the mouse models used and their age at the time of study which may affect outcome. Regardless of the outcomes, it is clear from these studies that manipulating RyR activity has profound effects on AD pathology across a range of symptoms and disease features.

In this series of experiments, we sought to determine if shorter-term, sub-chronic treatment (4 weeks) with the RyR inhibitor, dantrolene, is an effective strategy to normalize early pathogenic Ca^2+^ signaling and related synaptic transmission and plasticity deficits observed, as well as amyloid deposition, in the AD mice. By extension, we would expect similar effects in the human brain, and hope to prevent progression of AD pathology, rather than temporarily reduce symptoms. The origins of this approach are based on the knowledge that, in AD model systems, it is predominantly RyR-mediated responses that underlie aberrant intracellular Ca^2+^ signaling within synaptic compartments and contribute to altered synaptic homeostasis, and the observation that acute dantrolene *in vitro* can normalize ER Ca^2+^ signaling aberrations in presymptomatic AD mice [12]. More recently, and highly complementary to our findings, it has been demonstrated that extended chronic treatment (>10 months) with dantrolene reduced amyloid deposition and improved behavioral performance on learning and memory tasks [24]. Therefore, in this study, we treated AD and control mice at different stages of pathology (early, pre-histopathology without memory deficits, and mid-stage moderate plaque pathology coincident with onset of memory deficits) for a shorter duration with a nanocrystal formulation of dantrolene which crosses the blood-brain barrier [27]. We hypothesized that by normalizing an early pathogenic signaling cascade with far-reaching physiological consequences, we would stabilize a host of physiological, histopathological, and synaptic deficits. We measured evoked Ca^2+^ responses via ER sources (IP_3_R and RyR) and from spike-evoked Ca^2+^ entry, electrical membrane properties, RyR isoform expression, synaptic transmission and plasticity properties, and density of amyloid deposition. In sum, we found this approach to be highly effective in early and mid-stages of the disease, and propose that it could serve as a new approach for the development of effective, disease modifying therapeutic strategies to treat AD, and possibly other Ca^2+^-regulated neurodegenerative diseases.

An additional beneficial insight gleaned from this study is establishing the feed-forward association between ER Ca^2+^ upregulation and Aβ deposition, as normalizing RyR-evoked Ca^2+^ signaling significantly reduced the deposition of Aβ peptides and density of insoluble dense core plaques in mid-stage AD mice. It would be expected that earlier intervention would further reduce the extent of amyloid pathology and the related structural pathology associated with amyloid deposits.

Dantrolene is an attractive drug to consider for AD treatment in that it already is in clinical use for malignant hyperthermia and muscle spasticity, among other conditions. Since dantrolene is known to have multiple neuroprotective effects [42], modifying an existing drug for the prevention of AD progression would provide a much-needed breakthrough towards designing effective drug therapies for AD, which at present do not exist in this rapidly aging population. This opportunity is particularly exciting in light of the low success rate of past AD clinical trials. The compounds designed to clear Aβ via immunotherapy or inhibition of secretase function have failed to slow disease progression and in some cases worsened cognitive function as well as increased the risk of developing other diseases such as encephalitis and skin cancer [43]–[46]. Another fundamental problem with clinical trials involves the timing of treatment. In most cases, treatment begins when patients present with behavioral symptoms, at which point the brain is in a considerable state of degeneration. There is compelling evidence that early pathological mechanisms occur long before clinical onset of AD [30], [47], thus requiring treating patients earlier in the disease, or even at presymptomatic stages, for treatment to be effective. As such, this form of dantrolene treatment when given at vulnerable yet measurable time points, such as a diagnosis of MCI or traumatic brain injury (TBI), may provide neuroprotective benefits such that subsequent synaptic pathology, histopathology, and cognitive loss are halted and possibly reversed.

The goal of this study was to examine whether sub-chronic treatment with the RyR antagonist dantrolene in AD mouse models would normalize ER Ca^2+^ signaling disruptions, reduce RyR2 expression, stabilize downstream synaptic transmission and plasticity expression, and reduce amyloid pathology. The findings are highly promising, particularly with the minimal effects observed in the NonTg mice and the profound therapeutic responses at both early and middle stages of the disease with modest (4 week) treatment. While encouraging overall, additional concerns and questions still need to be addressed prior to further considerations in a clinical population. While RyR2 is extensively expressed in AD-vulnerable brain regions, it is also highly expressed in cardiac muscle. While in the present study sub-chronic dantrolene treatment did not have any detectable adverse side effects on development, body weight or cardiac weight, these are features that must be watched closely in candidate patients for long-term dantrolene treatment. Further, dantrolene has differential inhibitory properties on Ca^2+^ release for the three RyR isoforms, with the relatively weakest effects on RyR2 [48]. Thus, a compound more specific for stabilizing RyR2 and targeted to the brain could be optimal for AD therapeutics. It is also important to consider the role of the RyR3, as it is upregulated at disease stages coincident with Aβ_1–42_ deposition, and is thought to be a potential neuroprotective response to amyloid exposure [49].

To advance AD drug discovery, there needs to be a shift in current research priorities to address newer concepts. This includes strategies that target the Ca^2+^ signaling disruptions which are imbedded in all the major features and risk factors for AD. Compounds that would normalize aberrant Ca^2+^ signaling could likely impede pathogenic cascades, and alter the course of the disease rather than merely delay the progression of cognitive symptoms.

Financial Disclosure: JCR is employed by R&D China, U.K Group, GlaxoSmithKline; BC and VGC are employed by Lyotropic Therapeutics.

## References

[1] Hardy J, Selkoe DJ (2002) The amyloid hypothesis of Alzheimer's disease: progress and problems on the road to therapeutics. Science 297: 353–356. 12130773 10.1126/science.1072994

[2] Aisen PS, Andrieu S, Sampaio C, Carrillo M, Khachaturian ZS, et al (2011) Report of the task force on designing clinical trials in early (predementia) AD. Neurology 76: 280–286. 21178097 10.1212/WNL.0b013e318207b1b9 PMC3034393

[3] Galimberti D, Scarpini E (2011) Disease-modifying treatments for Alzheimer's disease. Ther Adv Neurol Disord 4: 203–216. 21765871 10.1177/1756285611404470 PMC3131171

[4] Karran E, Mercken M, De Strooper B (2011) The amyloid cascade hypothesis for Alzheimer's disease: an appraisal for the development of therapeutics. Nat Rev Drug Discov 10: 698–712. 21852788 10.1038/nrd3505

[5] Selkoe DJ (2011) Resolving controversies on the path to Alzheimer's therapeutics. Nat Med 17: 1060–1065. 21900936 10.1038/nm.2460

[6] Goldman WP, Price JL, Storandt M, Grant EA, McKeel DW, et al (2001) Absence of cognitive impairment or decline in preclinical Alzheimer's disease. Neurology 56: 361–367. 11171902 10.1212/wnl.56.3.361

[7] Price JL, McKeel DW Jr, Buckles VD, Roe CM, Xiong C, et al (2009) Neuropathology of nondemented aging: presumptive evidence for preclinical Alzheimer disease. Neurobiol Aging 30: 1026–1036. 19376612 10.1016/j.neurobiolaging.2009.04.002 PMC2737680

[8] Schmitt FA, Davis DG, Wekstein DR, Smith CD, Ashford JW, et al (2000) "Preclinical" AD revisited: Neuropathology of cognitively normal older adults. Neurology 55: 370–376. 10932270 10.1212/wnl.55.3.370

[9] Coleman PD, Yao PJ (2003) Synaptic slaughter in Alzheimer's disease. Neurobiol Aging 24: 1023–1027. 14643374 10.1016/j.neurobiolaging.2003.09.001

[10] Scheff SW, Price DA (2006) Alzheimer's disease-related alterations in synaptic density: neocortex and hippocampus. J Alzheimers Dis 9: 101–115. 10.3233/jad-2006-9s312 16914849

[11] Oddo S, Caccamo A, Shepherd JD, Murphy MP, Golde TE, et al (2003) Triple-transgenic model of Alzheimer's disease with plaques and tangles: intracellular Abeta and synaptic dysfunction. Neuron 39: 409–421. 12895417 10.1016/s0896-6273(03)00434-3

[12] Stutzmann GE, Smith I, Caccamo A, Oddo S, Laferla FM, et al (2006) Enhanced ryanodine receptor recruitment contributes to Ca2+ disruptions in young, adult, and aged Alzheimer's disease mice. J Neurosci 26: 5180–5189. 16687509 10.1523/JNEUROSCI.0739-06.2006 PMC6674246

[13] Chakroborty S, Goussakov I, Miller MB, Stutzmann GE (2009) Deviant ryanodine receptor-mediated calcium release resets synaptic homeostasis in presymptomatic 3xTg-AD mice. J Neurosci 29: 9458–9470. 19641109 10.1523/JNEUROSCI.2047-09.2009 PMC6666542

[14] Goussakov I, Chakroborty S, Stutzmann GE (2011) Generation of dendritic Ca2+ oscillations as a consequence of altered ryanodine receptor function in AD neurons. Channels (Austin) 5: 9–13. 21139422 10.4161/chan.5.1.14124

[15] Goussakov I, Miller MB, Stutzmann GE (2010) NMDA-mediated Ca(2+) influx drives aberrant ryanodine receptor activation in dendrites of young Alzheimer's disease mice. J Neurosci 30: 12128–12137. 20826675 10.1523/JNEUROSCI.2474-10.2010 PMC2944253

[16] Chakroborty S, Kim J, Schneider C, Jacobson C, Molgo J, et al (2012) Early presynaptic and postsynaptic calcium signaling abnormalities mask underlying synaptic depression in presymptomatic Alzheimer's disease mice. J Neurosci 32: 8341–8353. 22699914 10.1523/JNEUROSCI.0936-12.2012 PMC3417348

[17] Cheung KH, Mei L, Mak DO, Hayashi I, Iwatsubo T, et al (2010) Gain-of-function enhancement of IP3 receptor modal gating by familial Alzheimer's disease-linked presenilin mutants in human cells and mouse neurons. Sci Signal 3: ra22. 20332427 10.1126/scisignal.2000818 PMC2898196

[18] Cheung KH, Shineman D, Muller M, Cardenas C, Mei L, et al (2008) Mechanism of Ca2+ disruption in Alzheimer's disease by presenilin regulation of InsP3 receptor channel gating. Neuron 58: 871–883. 18579078 10.1016/j.neuron.2008.04.015 PMC2495086

[19] Muller M, Cardenas C, Mei L, Cheung KH, Foskett JK (2011) Constitutive cAMP response element binding protein (CREB) activation by Alzheimer's disease presenilin-driven inositol trisphosphate receptor (InsP3R) Ca2+ signaling. Proc Natl Acad Sci U S A 108: 13293–13298. 21784978 10.1073/pnas.1109297108 PMC3156223

[20] Zhang H, Sun S, Herreman A, De Strooper B, Bezprozvanny I (2010) Role of presenilins in neuronal calcium homeostasis. J Neurosci 30: 8566–8580. 20573903 10.1523/JNEUROSCI.1554-10.2010 PMC2906098

[21] Kelliher M, Fastbom J, Cowburn RF, Bonkale W, Ohm TG, et al (1999) Alterations in the ryanodine receptor calcium release channel correlate with Alzheimer's disease neurofibrillary and beta-amyloid pathologies. Neuroscience 92: 499–513. 10408600 10.1016/s0306-4522(99)00042-1

[22] Supnet C, Noonan C, Richard K, Bradley J, Mayne M (2010) Up-regulation of the type 3 ryanodine receptor is neuroprotective in the TgCRND8 mouse model of Alzheimer's disease. J Neurochem 112: 356–365. 19903243 10.1111/j.1471-4159.2009.06487.x

[23] Stutzmann GE, Smith I, Caccamo A, Oddo S, Parker I, et al (2007) Enhanced ryanodine-mediated calcium release in mutant PS1-expressing Alzheimer's mouse models. Ann N Y Acad Sci 1097: 265–277. 17413028 10.1196/annals.1379.025

[24] Peng J, Liang G, Inan S, Wu Z, Joseph DJ, et al (2012) Dantrolene ameliorates cognitive decline and neuropathology in Alzheimer triple transgenic mice. Neurosci Lett 516: 274–279. 22516463 10.1016/j.neulet.2012.04.008 PMC3351794

[25] Oules B, Del Prete D, Greco B, Zhang X, Lauritzen I, et al (2012) Ryanodine Receptor Blockade Reduces Amyloid-beta Load and Memory Impairments in Tg2576 Mouse Model of Alzheimer Disease. J Neurosci 32: 11820–11834. 22915123 10.1523/JNEUROSCI.0875-12.2012 PMC3458216

[26] Howlett DR, Richardson JC, Austin A, Parsons AA, Bate ST, et al (2004) Cognitive correlates of Abeta deposition in male and female mice bearing amyloid precursor protein and presenilin-1 mutant transgenes. Brain Res 1017: 130–136. 15261108 10.1016/j.brainres.2004.05.029

[27] Filbert M LE, Ballough G (2005) Neuroprotection for nerve agent-induced brain damage by blocking delayed calcium overload: a review. J Med CBR Def 3: 21.

[28] Howlett DR, Bowler K, Soden PE, Riddell D, Davis JB, et al (2008) Abeta deposition and related pathology in an APP×PS1 transgenic mouse model of Alzheimer’s disease. Histol Histopathol 23: 67–76. 17952859 10.14670/HH-23.67

[29] Stutzmann GE, Parker I (2005) Dynamic multiphoton imaging: a live view from cells to systems. Physiology (Bethesda) 20: 15–21. 15653835 10.1152/physiol.00028.2004

[30] Stutzmann GE (2007) The pathogenesis of Alzheimers disease is it a lifelong "calciumopathy"? Neuroscientist 13: 546–559. 17901262 10.1177/1073858407299730

[31] Stutzmann GE, Caccamo A, LaFerla FM, Parker I (2004) Dysregulated IP3 signaling in cortical neurons of knock-in mice expressing an Alzheimer's-linked mutation in presenilin1 results in exaggerated Ca2+ signals and altered membrane excitability. J Neurosci 24: 508–513. 14724250 10.1523/JNEUROSCI.4386-03.2004 PMC6729995

[32] Bruno AM, Huang JY, Bennett DA, Marr RA, Hastings ML, et al.. (2012) Altered ryanodine receptor expression in mild cognitive impairment and Alzheimer's disease. Neurobiol Aging 33: 1001 e1001–1006. 10.1016/j.neurobiolaging.2011.03.011 PMC3160507 21531043

[33] Demuro A, Parker I, Stutzmann GE (2010) Calcium signaling and amyloid toxicity in Alzheimer disease. J Biol Chem 285: 12463–12468. 20212036 10.1074/jbc.R109.080895 PMC2857063

[34] Ferreira IL, Bajouco LM, Mota SI, Auberson YP, Oliveira CR, et al (2012) Amyloid beta peptide 1–42 disturbs intracellular calcium homeostasis through activation of GluN2B-containing N-methyl-d-aspartate receptors in cortical cultures. Cell Calcium 51: 95–106. 22177709 10.1016/j.ceca.2011.11.008

[35] Itkin A, Dupres V, Dufrene YF, Bechinger B, Ruysschaert JM, et al (2011) Calcium ions promote formation of amyloid beta-peptide (1–40) oligomers causally implicated in neuronal toxicity of Alzheimer's disease. PLoS One 6: e18250. 21464905 10.1371/journal.pone.0018250 PMC3065491

[36] Kawahara M, Ohtsuka I, Yokoyama S, Kato-Negishi M, Sadakane Y (2011) Membrane Incorporation, Channel Formation, and Disruption of Calcium Homeostasis by Alzheimer's beta-Amyloid Protein. Int J Alzheimers Dis 2011: 304583. 21547225 10.4061/2011/304583 PMC3087492

[37] Pierrot N, Ghisdal P, Caumont AS, Octave JN (2004) Intraneuronal amyloid-beta1–42 production triggered by sustained increase of cytosolic calcium concentration induces neuronal death. J Neurochem 88: 1140–1150. 15009669 10.1046/j.1471-4159.2003.02227.x

[38] Querfurth HW, Selkoe DJ (1994) Calcium ionophore increases amyloid beta peptide production by cultured cells. Biochemistry 33: 4550–4561. 8161510 10.1021/bi00181a016

[39] Avila J, Perez M, Lim F, Gomez-Ramos A, Hernandez F, et al (2004) Tau in neurodegenerative diseases: tau phosphorylation and assembly. Neurotox Res 6: 477–482. 15639780 10.1007/BF03033284

[40] Demuro A, Smith M, Parker I (2011) Single-channel Ca(2+) imaging implicates Abeta1–42 amyloid pores in Alzheimer's disease pathology. J Cell Biol 195: 515–524. 22024165 10.1083/jcb.201104133 PMC3206345

[41] Pierrot N, Santos SF, Feyt C, Morel M, Brion JP, et al (2006) Calcium-mediated transient phosphorylation of tau and amyloid precursor protein followed by intraneuronal amyloid-beta accumulation. J Biol Chem 281: 39907–39914. 17085446 10.1074/jbc.M606015200

[42] Muehlschlegel S, Sims JR (2009) Dantrolene: mechanisms of neuroprotection and possible clinical applications in the neurointensive care unit. Neurocrit Care 10: 103–115. 18696266 10.1007/s12028-008-9133-4 PMC2702250

[43] Extance A (2010) Alzheimer's failure raises questions about disease-modifying strategies. Nat Rev Drug Discov 9: 749–751. 20885394 10.1038/nrd3288

[44] Orgogozo JM, Gilman S, Dartigues JF, Laurent B, Puel M, et al (2003) Subacute meningoencephalitis in a subset of patients with AD after Abeta42 immunization. Neurology 61: 46–54. 12847155 10.1212/01.wnl.0000073623.84147.a8

[45] Sambamurti K, Greig NH, Utsuki T, Barnwell EL, Sharma E, et al (2011) Targets for AD treatment: conflicting messages from gamma-secretase inhibitors. J Neurochem 117: 359–374. 21320126 10.1111/j.1471-4159.2011.07213.x PMC3076515

[46] Gilman S, Koller M, Black RS, Jenkins L, Griffith SG, et al (2005) Clinical effects of Abeta immunization (AN1792) in patients with AD in an interrupted trial. Neurology 64: 1553–1562. 15883316 10.1212/01.WNL.0000159740.16984.3C

[47] Holtzman DM, Goate A, Kelly J, Sperling R (2011) Mapping the road forward in Alzheimer's disease. Sci Transl Med 3: 114 ps148. 10.1126/scitranslmed.3003529 22190237

[48] Zhao F, Li P, Chen SR, Louis CF, Fruen BR (2001) Dantrolene inhibition of ryanodine receptor Ca2+ release channels. Molecular mechanism and isoform selectivity. J Biol Chem 276: 13810–13816. 11278295 10.1074/jbc.M006104200

[49] Supnet C, Grant J, Kong H, Westaway D, Mayne M (2006) Amyloid-beta-(1–42) increases ryanodine receptor-3 expression and function in neurons of TgCRND8 mice. J Biol Chem 281: 38440–38447. 17050533 10.1074/jbc.M606736200

